# Modelling Entropy in Magnetized Flow of Eyring–Powell Nanofluid through Nonlinear Stretching Surface with Chemical Reaction: A Finite Element Method Approach

**DOI:** 10.3390/nano12111811

**Published:** 2022-05-25

**Authors:** Salman Saleem, Degavath Gopal, Nehad Ali Shah, Nosheen Feroz, Naikoti Kishan, Jae Dong Chung, Saleha Safdar

**Affiliations:** 1 Department of Mathematics, College of Science, King Khalid University, Abha 61413, Saudi Arabia; saakhtar@kku.edu.sa; 2 Department of Mathematics, KG Reddy College of Engineering and Technology, Hyderabad 500075, India; degavathgopal@gmail.com; 3 Department of Mechanical Engineering, Sejong University, Seoul 05006, Korea; jdchung@sejong.ac.kr; 4 Department of Mathematics, Bacha Khan University, Charsadda P.O. Box 20, Pakistan; nosheen26@yahoo.com; 5 Department of Mathematics, Osmania University, Hyderabad 500007, India; nkishan@gmail.co; 6 Independent Researcher, Islamabad 44000, Pakistan; salehasafdar@gmail.com

**Keywords:** Bejan number, chemical reaction, entropy, Eyring–Powell fluid, finite element method

## Abstract

The present paper explores the two-dimensional (2D) incompressible mixed-convection flow of magneto-hydrodynamic Eyring–Powell nanofluid through a nonlinear stretching surface in the occurrence of a chemical reaction, entropy generation, and Bejan number effects. The main focus is on the quantity of energy that is lost during any irreversible process of entropy generation. The system of entropy generation was examined with energy efficiency. The set of higher-order non-linear partial differential equations are transformed by utilizing non-dimensional parameters into a set of dimensionless ordinary differential equations. The set of ordinary differential equations are solved numerically with the help of the finite element method (FEM). The illustrative set of computational results of Eyring–Powell (E–P) flow on entropy generation, Bejan number, velocity, temperature, and concentration distributions, as well as physical quantities are influenced by several dimensionless physical parameters that are also presented graphically and in table-form and discussed in detail. It is shown that the Schemit number increases alongside an increase in temperature, but the opposite trend occurs in the Prandtl number. Bejan number and entropy generation decline with the effect of the concentration diffusion parameter, and the results are shown in graphs.

## 1. Introduction

The non-Newtonian fluids have wide application in engineering, technology, various sciences, the processing and devolatilization of polymers, and industrial processes. They also find essential use in processes within the chemical industry. Examples of non-Newtonian fluids include: paint, blood, liquid crystals, the thixotropic-like ink in a Fisher Space Pen, grease or lubricants, the gravity acting to provide lubrication under the shear force fluids, etc. The different types of non-Newtonian fluids can be categorized by consenting nonlinear stress–strain constitutive models like the Casson model [1], Carreau-Yasuda model [2], power-law model [3], cross model [4] and Maxwell model [5], but these types of models do not describe the individual features of rheological fluids. Eyring–Powell fluid is one type of non-Newtonian fluid with contained plasticity in its accumulation viscosity. Several researchers have deliberated the importance of non-Newtonian fluids [6,7,8,9,10].

Eyring and Powell first considered Eyring–Powell fluid [11] in the year 1944. The amount of energy is defined as entropy (an irretrievable loss of thermal energy) rather than characterizing the total system as entropy generation. It is concluded that the thermal system is affected by the irretrievable loss of energy which is enhanced in system entropy and declines the efficacy of the taken system. The second law of thermodynamics deals with entropy constriction and defines the concert of thermal mechanics, such as manufacture in industry, utilizing the process of wire coating in several fluid polymers, viscoelastic metals, the storage of energy, and heat performance in refrigerators, various air conditions and solar ponds. Bejan [12,13] suggested the rate of entropy generation to be geometrically minimized with the convection of a heat transfer. Khan et al. [14] considered the development of wire coating, utilizing polymer liquid in the permeable medium along with the influence of fluctuating viscosity, and using Joule heating rather than wire layer pressure-nature to interact with Eyring–Powell fluid.

Recently, several authors have encouraged work with Eyring–Powell fluid, such as Raju et al. [15], who studied the mechanism of the nonlinear thermal and heat transfer of Cattaneo–Christov heat flux on magneto-hydrodynamic Eyring–Powell fluid. Wubshet and Gosa [16] explored the Eyring–Powell nanofluid with the Catteneo–Christov mass flux and heat flux models due to a non-linear continuously moving surface with chemical and absorption/generation that was solved by the GFE method. Sindhu and Gireesha’s [17] studies illuminate the influence of using an aluminum nanofluid flow form on a microchannel, and Grashof and Eckert numbers show a decline in entropy generation which is numerically achieved by the RKF method. Olumide et al. [18] focused on analyzing the mass and heat transfers of electrically conducting in-folding-radiative Eyring–Powell fluid due to a channel with the consequence of a chemical reaction and generation/absorption of heat flow. Several authors have reported the importance of Eyring–Powell fluid [19,20,21,22,23,24,25,26].

At present, activation energy has played a key role in chemical reactions in comparison to the lowest energy required to change the reactants to the material. In this circumstance, the relation between chemical and mass transmission converges monotonously. This communication exists in liquid and mass transfers in the manufacturing process. The use of stimulation energy is associated with chemical engineering, thermal processes, and the dynamics of water and oil tinctures. The influence of chemical reactions and convective forms on the peristaltic flow of Eyring–Powell fluid was explored by Hayat at al. [27]. Dhlamini et al. [28] analyzed the impact of chemical reaction and activation energy on mixed convection nanofluid flow. The binary chemical reaction and activation energy influence on the magnetic flow of Eyring–Powell with the stagnation point flow was encouraged by Reddy et al. [29]. Several researchers have discussed the influence of chemical reactions and the nanofuid phenomenon [30,31,32,33,34,35,36].

The present article aims to address the moment of entropy generation and Bejan number on magnetic Eyring–Powell nanofluid flow due to a stretching surface. The significant influence of mixed convection, fluid parameters and the chemical reaction was also studied. Little literature is available on the topic of magneto-hydrodynamic Eyring–Powell fluid. For numerical solutions of a specified system of nonlinear equations the finite element method [37,38,39] is utilized.

## 2. Mathematical Formation

**Figure 1:**
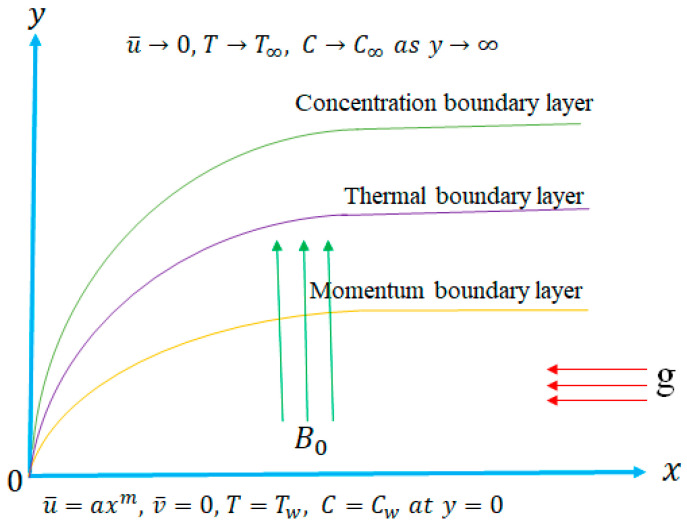
Flow geometry.

Assume a steady two-dimensional magneto-hydrodynamic Eyring–Powell nanofluid flow through a nonlinear stretching surface containing the influence of several physical parameters, such as Bejan number, energy generation, thermophoresis and Brownian motion. Consider the direction of velocity components and directions, respectively, where the non-linear stretching velocity is along the direction and stretching rate. The surface temperature concentration and ambient temperature concentration are represented, respectively. The strength of the constant magnetic parameter works parallel to the direction (see Figure 1). The involved governing equations are taken as [40,41,42].

(1) $$\frac{\partial \bar{u}}{\partial x}+\frac{\partial \bar{v}}{\partial y}=0$$

(2) $$\begin{array}{l} \bar{u}\frac{\partial \bar{u}}{\partial x}+\bar{v}\frac{\partial \bar{u}}{\partial y}=\left(\upsilon +\frac{1}{\rho_{f}\beta^{*}c_{1}}-\frac{1}{2\rho_{f}\beta^{*}c_{1}^{3}}{\left(\frac{\partial \bar{u}}{\partial y}\right)}^{2}\right)\frac{\partial^{2}\bar{u}}{\partial y^{2}}+g\left(\varepsilon_{1}(T-T_{\infty })+\varepsilon_{2}{\left(T-T_{\infty }\right)}^{2}\right)\;\\ +\;g\left(\varepsilon_{3}(C-C_{\infty })+\varepsilon_{4}{\left(C-C_{\infty }\right)}^{2}\right)-\frac{\sigma B_{0}^{2}}{\rho_{f}}\bar{u} \end{array}$$

(3) $$\begin{array}{l} \bar{u}\frac{\partial T}{\partial x}+\bar{v}\frac{\partial T}{\partial y}=\frac{k}{{\left(\rho c_{p}\right)}_{f}}\frac{\partial^{2}T}{\partial y^{2}}+\frac{1}{{\left(\rho c_{p}\right)}_{f}}\left(\frac{1}{\beta^{*}c_{1}}+\mu \right){\left(\frac{\partial \bar{u}}{\partial y}\right)}^{2}+\frac{1}{{\left(\rho c_{p}\right)}_{f}}\frac{1}{6\beta^{*}c_{1}^{3}}\;{\left(\frac{\partial \bar{u}}{\partial y}\right)}^{4}\\ +\frac{{\left(\rho c_{p}\right)}_{s}}{{\left(\rho c_{p}\right)}_{f}}\left(\frac{D_{T}}{T_{\infty }}{\left(\frac{\partial T}{\partial y}\right)}^{2}+D_{B}\frac{\partial C}{\partial y}\frac{\partial T}{\partial y}\right)+\frac{\sigma B_{0}^{2}}{{\left(\rho c_{p}\right)}_{f}}{\bar{u}}^{2} \end{array}$$

(4) $$\bar{u}\frac{\partial C}{\partial x}+\bar{v}\frac{\partial C}{\partial y}=D_{B}\frac{\partial^{2}C}{\partial y^{2}}+\frac{D_{T}}{T_{\infty }}\frac{\partial^{2}T}{\partial y^{2}}-K_{1}\left(C-C_{\infty }\right)$$

with consistent boundary conditions are

(5) $$\bar{u}={\bar{U}}_{w}=ax^{n},\;\bar{v}=0,\;T=T_{w},\;C=C_{w},\;\mathrm{at}\;y=0$$

$$\bar{u}\to 0,\;T\to T_{\infty },\;C\to C_{\infty },\;\mathrm{as}\;y\to \infty$$

Appropriate similarity transformations are

$$\bar{u}=ax^{n}F^{\prime }(\xi ),\;\bar{v}=-\;\sqrt{a\upsilon }F(\xi ),\;\xi =y\sqrt{a/\upsilon },\;\theta (\xi )=(T-T_{\infty })/(T_{w}-T_{\infty })$$

(6) $$\Phi (\xi )=(C-C_{\infty })/(C_{w}-C_{\infty })$$

After substituting the above transformation and identically satisfying Equation (1), Equations (2)–(6) are reduced as

(7) $$(1+\varepsilon )F^{\prime \prime \prime }-\varepsilon \delta {F^{\prime \prime }}^{2}F^{\prime \prime \prime }+\lambda \left(\left(\theta +\beta_{t}\theta^{2})+N^{*}(\Phi +\beta_{c}\Phi^{2}\right)\right)+FF^{\prime \prime }-{F^{\prime }}^{2}-MF^{\prime }=0$$

(8) $$\frac{1}{\mathrm{Pr}}\theta^{\prime \prime }+(1+\varepsilon )Ec{F^{\prime \prime }}^{2}-\frac{1}{3}\varepsilon \delta Ec{F^{\prime \prime }}^{4}+F\theta^{\prime }+N_{t}{\theta^{\prime }}^{2}+N_{b}\theta \Phi +EcM{F^{\prime }}^{2}=0$$

(9) $$\Phi^{\prime \prime }+ScF\Phi^{\prime }-ScC_{h}\Phi +(N_{t}/N_{b})\theta^{\prime \prime }=0$$

(10) $$\begin{array}{l} F(0)=0,\;F^{\prime }(0)=1,\;F^{\prime }(\infty )\to 0\\ \theta (0)=1,\Phi (0)=1,\;\theta (\infty )\to 0,\;\Phi (0)\to 0 \end{array}\}$$

Here, flow variables are defined as magnetic parameter is $M\left(=\sigma B_{0}^{2}/\rho a\right)$; ratio between concentration and thermal buoyancy force is $N^{*}\left(=Gr_{x}^{*}/Gr_{x}=\left(\varepsilon_{3}(C_{w}-C_{\infty })/\varepsilon_{1}(T_{w}-T_{\infty })\right)\right)$; Grashof number for temperature is $Gr_{x}\left(=g\varepsilon_{1}(T_{w}-T_{\infty })x^{3}/\upsilon^{2}\right)$; Grashof number for concentration is $Gr_{x}^{*}\left(=g\varepsilon_{3}(C_{w}-C_{\infty })x^{3}/\upsilon^{2}\right)$; non-linear mixed convection parameter of temperature is $\beta_{t}\left(=\varepsilon_{2}(T_{w}-T_{\infty })/\varepsilon_{1}\right)$; non-linear mixed convection parameter of concentration is $\beta_{c}\left(=\varepsilon_{4}(C_{w}-C_{\infty })/\varepsilon_{3}\right)$; mixed convection parameter λ(=Grx/Rex2); thermophoresis parameter is $N_{t}\left(={\left(\rho c_{p}\right)}_{s}D_{T}(T_{w}-T_{\infty })/{\left(\rho c_{p}\right)}_{f}\upsilon T_{\infty }\right)$; Brownian motion parameter is $N_{b}\left(={\left(\rho c_{p}\right)}_{s}D_{B}(C_{w}-C_{\infty })/{\left(\rho c_{p}\right)}_{f}\upsilon \right)$; dimensionless fluid parameters are $\varepsilon \left(=1/\mu \beta^{*}c_{1}\right)$, and $\delta \left(={\bar{U}}_{w}^{3}/2x\upsilon c_{1}^{2}\right)$; Prandtl number is $\mathrm{Pr}\left(=\upsilon {\left(\rho c_{p}\right)}_{f}/k\right)$; Schmidt number is $Sc\left(=\upsilon /D_{B}\right)$; Eckert number is $Ec\left(=a^{2}x^{2}/c_{p}(T_{w}-T_{\infty })\right)$; and Reynolds number is Rex(=U¯wx/υ).

The physical quantities are heat surface, mass transfer and drag force and can be represented as

(11) $$C_{f}\left(=\frac{\tau_{w}}{\rho {\bar{U}}_{w}^{2}}\right),Nu_{x}\left(=\frac{xq_{w}}{k(T_{w}-T_{\infty })}\right)\;,\;Sh_{x}\left(=\frac{xq_{m}}{D_{B}(C_{w}-C_{\infty })}\right)\;.$$

Here, $\tau_{w},\;q_{w}$ and $q_{m}$ are given as

(12) $$\tau_{w}{\left(=\left(\mu +\frac{1}{c_{1}\beta^{*}}\right)\frac{\partial \bar{u}}{\partial y}-\frac{1}{6c_{1}^{3}\beta^{*}}{\left(\frac{\partial \bar{u}}{\partial y}\right)}^{3}\right)}_{y=0}\;,\;q_{w}{\left(=-k\left(\frac{\partial T}{\partial y}\right)\right)}_{y=0},q_{m}{\left(=-D_{B}\left(\frac{\partial C}{\partial y}\right)\right)}_{y=0}$$

The non-dimensional parameters are the skin friction coefficient $\left(C_{f}\right)$, Nusselt number $\left(Nu_{x}\right)$ and Sherwood number $\left(Sh_{x}\right)$ taken as:

(13) CfRex1/2(=(1+ε)F″(0)−13εδ(F″(0))3), NuxRex−1/2(=−θ′(0)), ShxRex−1/2(=−Φ′(0))

Here Rex(=ax2υ) is a Reynolds number.

## 3. Entropy Generation

The entropy generation contains four factors: Joule dissipation, heat transfer, mass transfer and viscous dissipation. The entropy generation volumetric rate of viscous fluid for the magnetic and electric fields are obtained as [43,44,45,46]. The entropy equation for the Eyring–Powell fluid follows as

(14) $$S_{G}=\frac{k}{T_{\infty }^{2}}{\left(\frac{\partial T}{\partial y}\right)}^{2}+\frac{1}{T_{\infty }}\left(\left(\mu +\frac{1}{\beta^{*}c_{1}}\right){\left(\frac{\partial \bar{u}}{\partial y}\right)}^{2}-\frac{1}{6\beta^{*}c_{1}^{3}}{\left(\frac{\partial \bar{u}}{\partial y}\right)}^{4}\right)+\frac{R^{*}D}{C_{\infty }}{\left(\frac{\partial C}{\partial y}\right)}^{2}+\frac{R^{*}D}{T_{\infty }}\left(\frac{\partial T}{\partial y}\frac{\partial C}{\partial y}\right)+\frac{\sigma B_{0}^{2}}{T_{\infty }}{\bar{u}}^{2}$$

where the dimensionless volumetric entropy rate of generation owing the fluid friction and heat transfer of the form

(15) $$N_{G}=\alpha_{1}{\theta^{\prime }}^{2}+Br(1+\varepsilon ){F^{\prime \prime }}^{2}+L_{1}\frac{\alpha_{2}}{\alpha_{1}}{\Phi^{\prime }}^{2}+L_{1}\Phi^{\prime }\theta^{\prime }-\frac{1}{3}Br\varepsilon \delta {F^{\prime \prime }}^{4}+MBr{F^{\prime }}^{2}$$

Here, the dimensionless parameters represented as the temperature difference parameter is $\alpha_{1}\left(=\Delta T/T_{\infty }\right)=(T_{w}-T_{\infty })/T_{\infty }$; the concentration difference parameter is $\alpha_{2}\left(=\Delta C/C_{\infty }\right)=(C_{w}-C_{\infty })/C_{\infty }$; the diffusion parameter is $L_{1}\left(=R^{*}D(C_{w}-C_{\infty })/k\right)$; local entropy generation is $N_{G}\left(=T_{\infty }S_{G}\upsilon /k\Delta T\right)$; and Brinkman number is $Br\left(=\mu a^{2}x^{2}/k\Delta T\right)$.

The Bejan number (Be) is formed as

(16) $$Be=\frac{\alpha_{1}{\theta^{\prime }}^{2}+L_{1}(\alpha_{2}/\alpha_{1}){\Phi^{\prime }}^{2}+L_{1}\Phi^{\prime }\theta^{\prime }}{\alpha_{1}{\theta^{\prime }}^{2}+Br(1+\varepsilon ){F^{\prime \prime }}^{2}+L_{1}\frac{\alpha_{2}}{\alpha_{1}}{\Phi^{\prime }}^{2}+L_{1}\Phi^{\prime }\theta^{\prime }-\frac{1}{3}Br\varepsilon \delta {F^{\prime \prime }}^{4}+MBr{F^{\prime }}^{2}}$$

## 4. FEM Solution

The third order dimensionless differential equation is transformed into a second order dimensionless differential equation by using $F^{\prime }=h$. the dimensionless differential Equations (7)–(9) are written in residual form. These residuals are multiplied with weighted functions and integrated with a typical two node element $\left(\eta_{e},\;\eta_{e+1}\right)$ given by

(17) $$\int_{\eta_{e}}^{\eta_{e+1}}w_{t}\left\{F^{\prime }-h\right\}d\xi =0,$$

(18) $$\int_{\eta_{e}}^{\eta_{e+1}}w_{t}\left\{\left(1+\varepsilon \right)h^{\prime \prime }-\varepsilon \delta {\left(h^{\prime }\right)}^{2}h^{\prime \prime }+Fh^{\prime }+\lambda (\theta +\beta_{t}\theta^{2})+\lambda N^{*}(\Phi +\beta_{c}\Phi^{2})-h^{2}-M\;h\right\}d\xi =0,$$

(19) $$\int_{\eta_{e}}^{\eta_{e+1}}w_{t}\left\{\frac{1}{\mathrm{Pr}}\theta^{\prime \prime }+(1+\varepsilon )Ec{\left(h^{\prime }\right)}^{2}-\frac{1}{3}\varepsilon \delta Ec{\left(h^{\prime }\right)}^{4}+F\theta^{\prime }+N_{t}{\left(\theta^{\prime }\right)}^{2}+N_{b}\Phi \theta +M\;Ec\;h^{2}\right\}d\xi =0,$$

(20) $$\int_{\eta_{e}}^{\eta_{e+1}}w_{t}\left\{\Phi^{\prime \prime }+ScF\Phi^{\prime }+\frac{N_{t}}{N_{b}}\theta^{\prime \prime }-Sc\;C_{h}\Phi \right\}d\xi =0,$$

where $w_{t}$, (t=1,2,3,4) are weight functions and the variational functions are taken as F, h, θ, Φ, respectively. The unknown functions are approached by Galerkin approximations. The finite element model Equations (17)–(20) are achieved by substituting of finite element approximation form.

(21) $$F=\sum_{j=1}^{2}F_{j}\psi_{j},\;\;h=\sum_{j=1}^{2}h_{j}\psi_{j},\;\theta =\sum_{j=1}^{2}\theta_{j}\psi_{j},\;\Phi =\sum_{j=1}^{2}\Phi_{j}\psi_{j}$$

The unknown nodal values are $F_{j},\;h_{j},\;\theta_{j}$, $\Phi_{j}$ and the linear shape function for $\psi_{j}$, a typical line element $\left(\eta_{e},\eta_{e+1}\right)$ are presented as,

(22) $$\psi_{1}=\frac{\xi_{e+1}-\xi }{\xi_{e+1}-\xi_{e}},\;\psi_{2}=\frac{\xi -\xi_{e}}{\xi_{e+1}-\xi_{e}},\;\xi_{e}\leq \xi \leq \xi_{e+1}.$$

The model of the finite element equation is expressed as

(23) $$\left[\begin{array}{cccc} \left[K^{11}\right] & \left[K^{12}\right] & \left[K^{13}\right] & \left[K^{14}\right]\\ \left[K^{21}\right] & \left[K^{22}\right] & \left[K^{23}\right] & \left[K^{24}\right]\\ \left[K^{31}\right] & \left[K^{32}\right] & \left[K^{33}\right] & \left[K^{34}\right]\\ \left[K^{41}\right] & \left[K^{42}\right] & \left[K^{43}\right] & \left[K^{44}\right] \end{array}\right]\left[\begin{array}{c} \left\{F^{}\right\}\\ \left\{h^{}\right\}\\ \left\{\theta^{}\right\}\\ \left\{\Phi^{}\right\} \end{array}\right]=\left[\begin{array}{c} \left\{b^{1}\right\}\\ \left\{b^{2}\right\}\\ \left\{b^{3}\right\}\\ \left\{b^{4}\right\} \end{array}\right]$$

The matrices of $\left[K^{mn}\right]$ and $\left[b^{m}\right]$(m,n=1,2,3,4) are defined as

$$K_{ij}^{11}=\int_{\eta_{e}}^{\eta_{e+1}}\psi_{i}\frac{\partial \psi_{j}}{\partial \xi }d\xi ,\;K_{ij}^{12}=-\int_{\eta_{e}}^{\eta_{e+1}}\psi_{i}\psi_{j}d\xi ,\;K_{ij}^{13}=K_{ij}^{14}=0,\;K_{ij}^{21}=0,$$

$$\begin{array}{l} K_{ij}^{22}=-\left(1+\varepsilon \right)\int_{\eta_{e}}^{\eta_{e+1}}\frac{\partial \psi_{i}}{\partial \xi }\frac{\partial \psi_{j}}{\partial \xi }d\xi +\varepsilon \delta \left(\int_{\eta_{e}}^{\eta_{e+1}}{\left(\bar{h^{\prime }}\right)}^{2}\frac{\partial \psi_{i}}{\partial \xi }\frac{\partial \psi_{j}}{\partial \xi }d\xi \right)+\int_{\eta_{e}}^{\eta_{e+1}}\bar{F}\psi_{i}\frac{\partial \psi_{j}}{\partial \xi }d\xi \\ -\int_{\eta_{e}}^{\eta_{e+1}}\bar{h}\psi_{i}\psi_{j}d\xi -M\int_{\eta_{e}}^{\eta_{e+1}}\psi_{i}\psi_{j}d\xi , \end{array}$$

$$K_{ij}^{23}=\lambda \left[\int_{\eta_{e}}^{\eta_{e+1}}\psi_{i}\psi_{j}d\xi +\beta_{t}\int_{\eta_{e}}^{\eta_{e+1}}\bar{\theta }\psi_{i}\psi_{j}d\xi \right],\;K_{ij}^{24}=\lambda N^{*}\left[\int_{\eta_{e}}^{\eta_{e+1}}\psi_{i}\psi_{j}d\xi +\beta_{c}\int_{\eta_{e}}^{\eta_{e+1}}\bar{\Phi }\psi_{i}\psi_{j}d\xi \right],$$

$$\;K_{ij}^{31}=0,\;K_{ij}^{34}=0,\;$$

$$K_{ij}^{32}=(1+\varepsilon )Ec\int_{\eta_{e}}^{\eta_{e+1}}\bar{h^{\prime }}\psi_{i}\frac{\partial \psi_{j}}{\partial \xi }d\xi -\frac{1}{3}\varepsilon \delta Ec\int_{\eta_{e}}^{\eta_{e+1}}{\left(\bar{h^{\prime }}\right)}^{3}\psi_{i}\frac{\partial \psi_{j}}{\partial \xi }d\xi +MEc\int_{\eta_{e}}^{\eta_{e+1}}\bar{h}\psi_{i}\psi_{j}d\xi ,$$

$$K_{ij}^{33}=-\frac{1}{\mathrm{Pr}}\int_{\eta_{e}}^{\eta_{e+1}}\frac{\partial \psi_{i}}{\partial \xi }\frac{\partial \psi_{j}}{\partial \xi }d\xi +\int_{\eta_{e}}^{\eta_{e+1}}\bar{F}\psi_{i}\frac{\partial \psi_{j}}{\partial \xi }d\xi +N_{t}\int_{\eta_{e}}^{\eta_{e+1}}\bar{\theta^{\prime }}\psi_{i}\frac{\partial \psi_{j}}{\partial \xi }d\xi +N_{b}\int_{\eta_{e}}^{\eta_{e+1}}\bar{\Phi }\psi_{i}\psi_{j}d\xi ,$$

$$K_{ij}^{41}=K_{ij}^{42}=0,\;K_{ij}^{43}=\frac{N_{t}}{N_{b}}\int_{\eta_{e}}^{\eta_{e+1}}\frac{\partial \psi_{i}}{\partial \xi }\frac{\partial \psi_{j}}{\partial \xi }d\xi$$

(24) $$K_{ij}^{44}=-\int_{\eta_{e}}^{\eta_{e+1}}\frac{\partial \psi_{i}}{\partial \xi }\frac{\partial \psi_{j}}{\partial \xi }d\xi +Sc\int_{\eta_{e}}^{\eta_{e+1}}\bar{F}\psi_{i}\frac{\partial \psi_{j}}{\partial \xi }d\xi -ScC_{h}\int_{\eta_{e}}^{\eta_{e+1}}\psi_{i}\psi_{j}d\xi$$

(25) $$b_{i}^{1}=0,\;b_{i}^{2}=-\left((1+\varepsilon )+\varepsilon \delta {\left(\bar{h^{\prime }}\right)}^{2}\right){\left(\psi_{i}\frac{\partial h}{\partial \xi }\right)}_{\eta_{e}}^{\eta_{e+1}},\;b_{i}^{3}=-\frac{1}{\mathrm{Pr}}{\left(\psi_{i}\frac{\partial \theta }{\partial \xi }\right)}_{\eta_{e}}^{\eta_{e+1}},\;b_{i}^{4}=-{\left(\psi_{i}\frac{\partial \Phi }{\partial \xi }\right)}_{\eta_{e}}^{\eta_{e+1}}$$

where

$$\bar{F}=\sum_{i=1}^{2}\bar{F}\psi_{i},\;\bar{h}=\sum_{i=1}^{2}\bar{h}\psi_{i},\;\bar{\theta }=\sum_{i=1}^{2}\bar{\theta }\psi_{i},\;\bar{\Phi }=\sum_{i=1}^{2}\bar{\Phi }\psi_{i}$$

The whole domain is divided into 500 linear elements which are of equivalent sizes. It is solved iteratively. The three functions $F^{\prime },\;\theta ,\;\Phi$ are examined at each node. The assumed known functions are $\bar{F},\;\bar{h},\;\bar{\theta }$ and $\bar{\Phi }$, and are used for the linearized system. The velocity, temperature and concentration are set as equal to one for the first iteration. This process is repeated until the accuracy value is $10^{-5}$. The convergence results are calculated as the number of elements for n=10, 20, 40, 80, 160, 320, 400 and 500. The convergent results are shown in Table 1.

## 5. Results and Discussion

Here, the aspects of the non-dimensional governing equations and the corresponding boundary conditions are solved numerically using the finite element method (FEM). The numerical solution and results that are carried out for the influence of non-dimensional flow parameters are M=1.3, $N^{*}=0.2,$ λ=0.5, δ=0.9, $\beta_{t}=1,\;$$\beta_{c}=1.5,$ Ec=0.2, Pr=1.73, $N_{t}=0.3,\;$Sc=1, $N_{b}=0.8,\;$$C_{h}=0.08$ on Bejan number (Be); entropy generation $\left(N_{G}\right)$; velocity $\left(F^{\prime }(\xi )\right)$; temperature (θ(ξ)); and concentration (Φ(ξ)) distributions. Numerical results of skin friction $\left(C_{f}\right)$, Nusselt number $\left(Nu_{x}\right)$, and Sherwood number $\left(Sh_{x}\right)$ are presented in Table 2.

**Figure 2:**
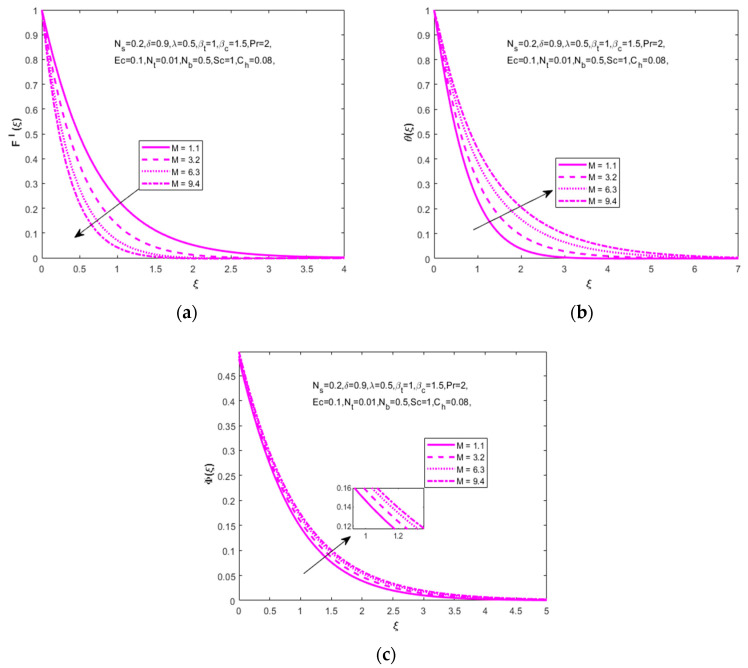
Effect of magnetic parameter M on (**a**) velocity $(F^{\prime }(\xi )),$ (**b**) temperature (θ(ξ)) and (**c**) concentration (Φ(ξ)), respectively.

Figure 2a–c express the influence of a magnetic parameter (M=1.1, 3.2, 6.3, 9.4) on velocity $\left(F^{\prime }(\xi )\right)$, temperature (θ(ξ)), concentration (Φ(ξ)) distributions. In Figure 2a it is observed that the velocity boundary layer thickness decreases with increased values of the magnetic parameter. This occurs when the expanding values of the magnetic parameter magnify the Lorentz force which resists the fluid motion and intensity in the velocity profile. Figure 2b,c shows that the behavior of a magnetic parameter on temperature and concentration distributions increases with the increased values of the magnetic parameter.

**Figure 3:**
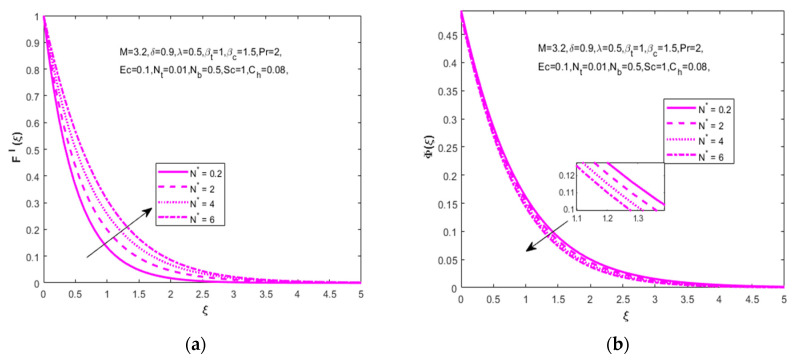
Impact of approximation parameter $N^{*}$ on (**a**) velocity $\left(F^{\prime }(\xi )\right)$ and (**b**) concentration (Φ(ξ)), respectively.

**Figure 4:**
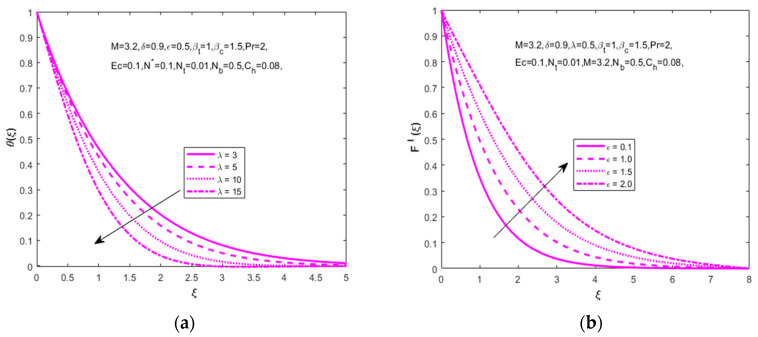
Impact of mixed convection parameter λ on (**a**) temperature (θ(ξ)), fluid parameter ε on (**b**) velocity $\left(F^{\prime }(\xi )\right)$, respectively.

Impact of $N^{*}$ on velocity $\left(F^{\prime }(\xi )\right)$ and concentration (Φ(ξ)) distributions are captured in Figure 3a,b. For the higher approximation of $N^{*}(0.2,\;2,\;4,\;6)$ the velocity distribution increases and the concentration distribution declines, meaning that both types work in opposing ways. The mixed convection parameter λ(3,  5, 10, 15) influence on temperature distribution (θ(ξ)) is revealed in Figure 4a. It is observed that an enlarged mixed convection parameter causes an identical slight decline in the temperature distribution. The dimensionless fluid parameter’s impact on velocity distribution $\left(F^{\prime }(\xi )\right)$ leads to a higher estimation of the non-dimensional fluid parameter ε(0.1, 1.0, 1.5, 2.0). How to enlarge the velocity distribution is shown in Figure 4b.

**Figure 5:**
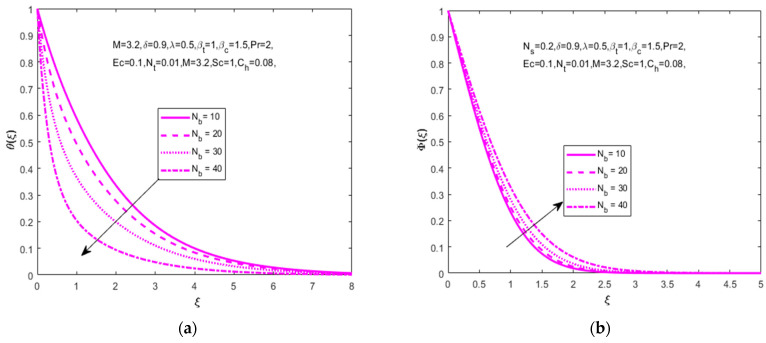
Impact of Brownian motion parameter $N_{b}$ on (**a**) temperature (θ(ξ)) and (**b**) concentration Φ(ξ), respectively.

**Figure 6:**
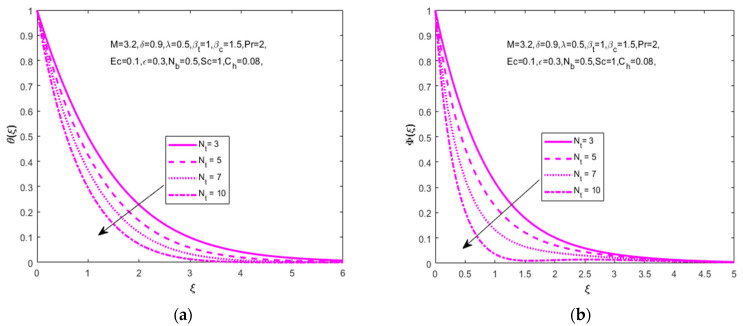
Impact of thermophoresis parameter $N_{t}$ on (**a**) temperature θ(ξ) and (**b**) concentration Φ(ξ), respectively.

The influence of $\left(N_{b}\right)$ is analyzed in Figure 5a,b for several values of the Brownian motion parameter $\left(N_{b}=10,\;20,\;30,\;40\right)$ on temperature distribution (θ(ξ)), and declines in concentration distribution (Φ(ξ)) that need to be increased. The Brownian motion parameter plays a key role on the surrounding liquids during the heat transfer. Figure 6a,b depicts that the increased values of the thermophoresis parameter $\left(N_{t}=3,\;5,\;7,\;10\right)$ are produced in temperature (θ(ξ)) and concentration (Φ(ξ)) distributions. An enhancement of thermophoresis parameters causes an increment in the force of thermophoresis. This is a decline in temperature (θ(ξ)) and concentration (Φ(ξ)) distributions.

**Figure 7:**
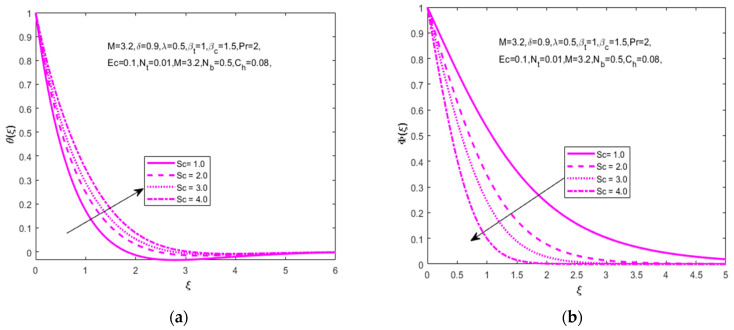
Impact of Schmidt number Sc on (**a**) temperature θ(ξ) and (**b**) concentration Φ(ξ), respectively.

**Figure 8:**
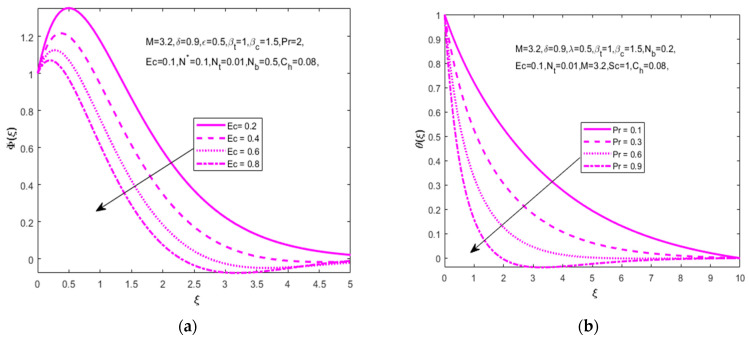
Impact of Eckert number Ec on (**a**) concentration Φ(ξ) and Prandtl number Pr on (**b**) temperature θ(ξ), respectively.

Figure 7a,b explain the variation in temperature (θ(ξ)) and concentration (Φ(ξ)) distributions for the increasing values of the Schmidt number (Sc=1.0, 2.0, 3.0, 4.0). The formulation of the Schmidt number is the ratio of the viscous diffusivity rate to the molecular diffusivity rate. The increasing values of the Schmidt number lowers the rate of molecular diffusion and sources the diffusion of higher density species in air. Here, we achieve the results of an increase in temperature distribution (θ(ξ)) and a decrease in fluid concentration distribution (Φ(ξ)). Figure 8a shows that the increasing values of the Eckert number (Ec=0.2, 0.4, 0.6, 0.8) with the concentration distribution (Φ(ξ)) have declined.

**Figure 9:**
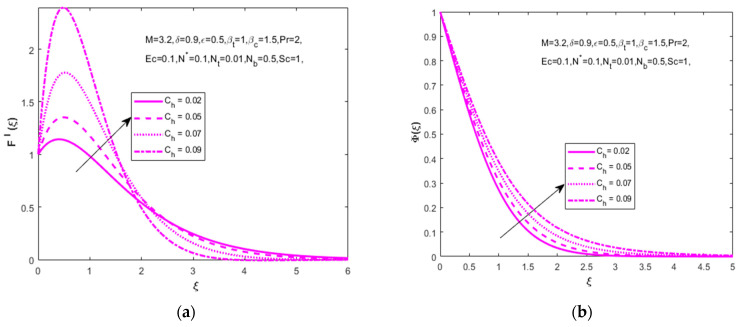
Impact of chemical reaction parameter $C_{h}$ on (**a**) velocity $\left(F^{\prime }(\xi )\right)$ and (**b**) concentration (Φ(ξ)), respectively.

The temperature distribution described in Figure 8b shows that the greatest values of the Prandtl number decrease the temperature distribution (θ(ξ)) due to the inverse properties of the thermal and Prandtl numbers. Figure 9a,b signifies the influence of the chemical reaction parameter. The important note is that the enhanced homogeneous chemical reaction parameter enhances the velocity $\left(F^{\prime }(\xi )\right)$ and concentration (Φ(ξ)) distributions.

**Figure 10:**
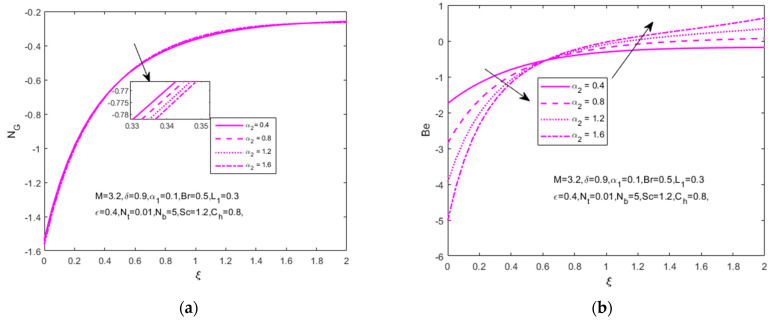

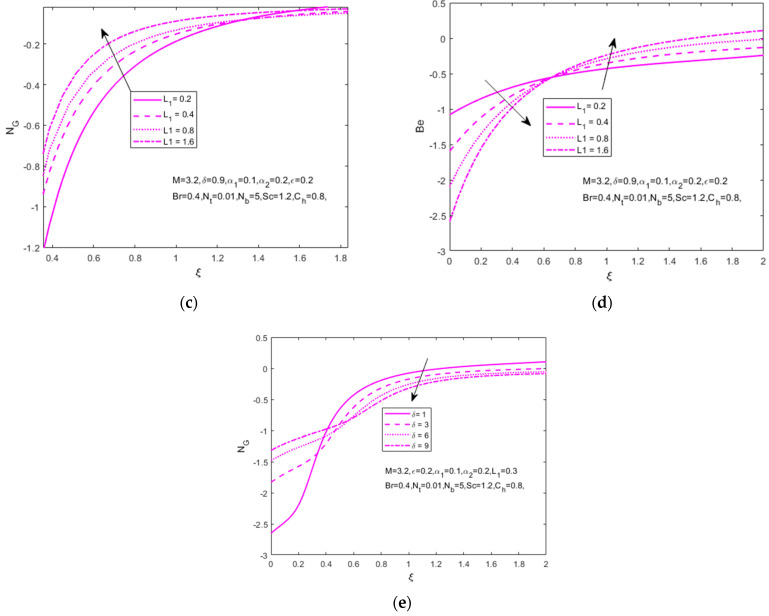
Impact of (**a**) difference parameter $\alpha_{2}$, (**b**) dimensionless parameter $L_{1}$ and fluid parameter δ on (**c**,**d**) entropy generation $\left(N_{G}(\xi )\right)$ and (**e**) Bejan number (Be(ξ)), respectively.

The influence of the concentration difference parameter $\left(\alpha_{2}=0.4,\;0.8,1.2,1.6\right)$, dimensionless parameter $\left(L_{1}=0.2,\;0.4,0.8,1.6\right)$, and fluid parameter δ(1,  3,6,9) on the entropy generation and Bejan number are shown in Figure 10a–e. The degree of energy generation $\left(N_{G}\right)$ decreases at a stretching surface where the concentration gradient increases. The Bejan number (Be) decreases at one frequency surface and increases towards the other frequency where a reaction occurs in the internal heat and fluid viscosity.

The increased values of $L_{1}$ thatt increase the entropy generation $\left(N_{G}\right)$ and Bejan number (Be) decrease one surface of frequency after increasing another frequency. This is because a higher irreversible diffusion parameter increases the rate of nanomaterials. Entropy generation $\left(N_{G}\right)$ decreases with the impact of an increased fluid parameter (δ).

Table 2 describes the numerical values of skin friction (CfRex1/2), Nusselt number (NuxRex−1/n+1) and Sherwood number (ShxRex−1/n+1) for various physical parameter effects. The measured skin friction increases for the mixed convection parameter (λ), fluid parameter (δ), buoyancy force parameter $\left(N^{*}\right)$ and chemical reaction parameter $\left(C_{h}\right)$ and decreases for the fluid parameter (ε), thermophorasis $\left(N_{t}\right)$ and Brownian motion parameter $\left(N_{b}\right)$. The Nusselt number increases for λ, δ, $N^{*}$, and the reverse process occurs with ε, $N_{t}$, $N_{b}$, $C_{h}$. The Sherwood number decreases for δ, ε, $N_{t}$ and increases with the mixed convection parameter (λ), buoyancy force parameter $\left(N^{*}\right)$, Brownian parameter $\left(N_{b}\right)$ and chemical parameter $\left(C_{h}\right)$.

## 6. Conclusions

The key points of this study are listed below.

▪ The Bejan number (Be) shows a decreasing influence for larger values of the dimensionless parameter $\left(L_{1}\right)$ and concentration diffusion parameter $\left(\alpha_{2}\right)$.
▪ Entropy generation $\left(N_{G}\right)$ displays a decreasing influence of $\left(\alpha_{2}\right)$, (δ) and an increasing influence with $\left(L_{1}\right)$.
▪ Skin friction $\left(C_{f}\right)$ showed reduced behavior for increasing values of (ε), $\left(N_{t}\right)$ and $\left(N_{b}\right)$, and inverse behavior with (λ), (δ), $\left(N^{*}\right)$ and $\left(C_{h}\right)$.
▪ The Nusselt number $\left(Nu_{x}\right)$ increased for the buoyancy force parameter $\left(N^{*}\right)$, fluid parameter (δ) and mixed convection parameter (λ).
▪ The Sherwood number $\left(Sh_{x}\right)$ decreased for the fluid parameter (δ), thermophorasis parameter $\left(N_{t}\right)$ and fluid parameter (ε).

## Figures and Tables

**Table 1:** Convergence of results exist at M=3.2, $N^{*}=0.5,$ ε=0.2, $\lambda =\beta_{t}=\beta_{c}=0.1,$ δ=0.9, $N_{t}=0.01,$ $N_{b}=5,$ Sc=1, $C_{h}=0.8$.

Number of Elements	*f* (2.4)	*θ* (2.4)	Φ (2.4)
10	0.4115	−0.0284	0.0143
20	0.4754	−0.0049	0.0151
40	0.4918	−0.0016	0.0153
80	0.4959	−0.0007	0.0154
160	0.4970	−0.0003	0.0154
320	0.4972	−0.0002	0.0154
400	0.4973	−0.0002	0.0154
500	0.4973	−0.0002	0.0154

**Table 2:** Numerical values of Skin friction, Nusselt number and Sherwood number are as follows.

λ	δ	ε	$N^{*}$	$N_{t}$	$N_{b}$	$C_{h}$	CfRex1/2	NuxRex−1/n+1	ShxRex−1/n+1
3	0.7	0.4	0.2	0.01	5	0.06	−1.2016	−4.2103	0.5117
5							−0.8539	−4.1688	0.5144
8							−0.3326	−4.1018	0.5182
	1.1						−1.6751	−4.2636	0.5073
	2.2						−1.4742	−4.2634	0.5050
	3.3						−1.2418	−4.2625	0.5019
		0.3					0.9156	−0.7570	0.4802
		0.6					0.5108	−0.8081	0.4707
		0.9					0.3409	−0.8675	0.4620
			0.1				−1.4414	−3.9755	0.5218
			0.3				−1.3104	−3.8265	0.5272
			0.5				−1.0557	−3.5509	0.5360
				2			−1.7167	−1.5205	0.3393
				4			−1.7194	−2.5978	0.1531
				8			−1.7226	−2.6844	0.0361
					5		−1.7204	−4.6232	0.0573
					10		−1.7214	−6.3809	0.2759
					20		−1.7227	−8.1039	0.3883
						0.02	−1.7229	−9.3079	0.8828
						0.04	−1.7228	−9.4305	0.9408
						0.08	−1.7227	−9.6344	1.0374

## Data Availability

The data used to support the findings of this study are included within the article.

## Nomenclature

u, v	Velocity-components in x− and y − directions
ν	Kinematic viscosity
T	Temperature
ρ	Density of the base fluid
${Τ}_{\infty }$	Constant temperature of the fluid
$N_{G}$	Local entropy generation
M	Magnetic parameter
Br	Brinkman number
$N^{*}$	Ratio between concentration and thermal buoyancy force is,
λ	Mixed convection parameter
$Gr_{x}$	Grashof number
Pr	Prandtl number
$\beta_{t}$	Non-linear mixed convection parameter
$N_{t}$	Thermophoresis parameter
$N_{b}$	Brownian motion parameter
Ec	Eckert number
ε	Dimensionless fluid parameter
Sc	Schmidt number
